# HLA-B27 Negativity Is Associated With Renal Function Decline in Patients With Ankylosing Spondylitis and Secondary IgA Nephropathy

**DOI:** 10.3389/fmed.2020.00089

**Published:** 2020-04-07

**Authors:** Ti Zhang, Fan Yang, Ke Zuo, Jinquan Wang, Zhen Cheng, Jiong Zhang

**Affiliations:** National Clinical Research Center of Kidney Diseases, Jinling Hospital, Nanjing University School of Medicine, Nanjing, China

**Keywords:** ankylosing spondylitis, IgA nephropathy, HLA-B27, outcome, pathogenesis

## Abstract

This study aimed to determine the impact of HLA-B27 on clinical phenotype and renal function during follow-up periods in patients with ankylosing spondylitis (AS) and secondary IgA nephropathy (IgAN). This single-center retrospective study included 71 AS patients with secondary IgAN. Renal function decline was defined as a mean eGFR decline of more than 5 mL/min/1.73 m^2^ per year or progression into the dialysis stage. The association between HLA-B27 status and renal function decline was evaluated by univariable and multivariable Cox regression analyses. The results showed that seven (9.85%) of the 71 included patients were HLA-B27-negative. The median follow-up period was 4.0 years. HLA-B27-negative patients showed higher levels of uric acid (UA) than those who were HLA-B27-positive. Pathologically, a higher percentage of globally sclerotic glomeruli was observed in HLA-B27-negative patients. Survival analysis indicated that HLA-B27 negativity was associated with a significantly higher probability of renal function decline than HLA-B27 positivity. This significant association was also found in subgroup analyses of patients with either substantial proteinuria (more than 1.0 g per day) or interstitial fibrosis and tubular atrophy. Multivariable analysis showed that HLA-27 negativity was independently associated with renal function decline (HR 6.58; 95% CI 1.65 to 26.21; *p* = 0.008). In conclusion, HLA-B27 negativity is associated not only with a higher level of UA and a higher percentage of globally sclerotic glomeruli in AS patients with secondary IgAN but with renal function decline during follow-up periods.

## Introduction

Ankylosing spondylitis (AS) is a chronic inflammatory disease predominantly affecting the spine, sacroiliac joints, and peripheral joints, which may ultimately lead to disability. In addition to extra-articular manifestations such as uveitis, psoriasis, and inflammatory bowel disease (IBD), some other organ systems may also be involved (1–3).

Renal involvement is an uncommon but significant complication that contributes to the poor prognosis of AS patients (4). The most common pathological type of renal lesions in AS is IgA nephropathy (IgAN), the diagnosis of which mainly depends on renal biopsy. Pathologically, IgAN is characterized by deposition of immune complexes in the glomerular mesangium, proliferation of mesangial cells, increased synthesis of extracellular matrix, and infiltration of proinflammatory cells (5). The clinical manifestations of IgAN include proteinuria, microscopic hematuria, and hypertension, alone or in combination. IgAN onset secondary to AS is evidenced by elevation of the serum level of IgA and mucosal infection, both of which are commonly seen in AS and IgAN patients (6). However, there is little knowledge about the clinical features and prognosis in this unique patient population owing to the requirement for pathological evidence.

The etiopathogenesis of AS is associated with the main genetic factor, HLA-B27, which is estimated to be present in 75–95% of AS cases. Previous studies reported that HLA-B27 was negativity associated with extra-articular manifestations, including psoriasis and IBD (7, 8). However, the association between HLA-B27 and AS with secondary IgAN has not been addressed. The current study was intended to describe the clinicopathological characteristics of AS with secondary IgAN and determine the link between the status of HLA-B27 and renal function decline during the follow-up period.

## Materials and Methods

### Patient Selection

Enrolled in this study were 71 AS patients who met the AS classification criteria established by the American College of Rheumatology (ACR) in 1984 in New York, with biopsy-proven IgAN registered in the Nanjing Glomerulonephritis Registry from 2007 to 2018. The exclusion criteria excluded AS patients with other autoimmune diseases, such as systemic lupus erythematosus and rheumatoid arthritis, and patients with a follow-up period of <12 months. The baseline and follow-up data of the patients were obtained from the database of the Nanjing Glomerulonephritis Registry. All follow-up data were updated to June 2019. This study was conducted in compliance with the Good Clinical Practice protocol and the Declaration of Helsinki principles and was approved by the Institutional Review Board of Jinling Hospital (Nanjing, China).

### Follow-Up Observation and Outcome Measures

The study included the following clinical and biological variables: age, gender, axial symptoms, peripheral involvement (peripheral arthritis and enthesitis), extra-articular manifestations (uveitis, IBD, psoriasis), the presence of HLA-B27, C-reactive protein (CRP), hemoglobin (Hb), platelet (PLT), uric acid (UA), serum creatinine (Scr), and 24 h urine protein secretion. Pathological parameters included the percentage of globally sclerotic glomeruli, focal segmental glomerular sclerosis, and interstitial fibrosis and tubular atrophy (IF/TA).

The follow-up period was defined as the time between the first visit (date of renal biopsy as the baseline) and the last available follow-up for each patient. The frequency of follow-up visits was at least twice a year. Laboratory testing was performed in all patients in every follow-up visit. The estimated glomerular filtration rate (eGFR) was estimated using the CKD-EPI formula. Renal function decline was defined as a mean eGFR decline > 5 mL/min/1.73 m^2^ per year or progression into the dialysis stage. The end-stage of renal disease (ESRD) was defined as eGFR <15 mL/min/1.73m^2^ and initiation of dialysis or transplantation.

### Statistical Methods

Group comparisons were made using Student's *t*-test, Chi-square test, or Fisher's exact test, as appropriate. Renal survival was analyzed by using Kaplan–Meier curves and the log-rank test. A *p*-value of < 0.05 was considered to indicate statistical significance. For multivariate regression models, all covariates with *P* ≤ 0.10 in the univariate analysis were included in the multivariate analysis. Data analyses were performed using SPSS software version 25.0 (SPSS, Inc., Chicago, IL, USA).

## Results

### Demographic and Clinical Characteristics

A total of 71 AS patients with biopsy-proven IgAN were included for analysis, of whom seven (9.85%), all of whom were male, had negative HLA-B27. The mean age of the patients in our series was 36.6 years, the mean AS duration was 11.8 years, and the median follow-up period was 4 years. No difference in articular or extra-articular manifestations was observed between the HLA-B27-negative and positive patients. Biomedical tests showed that HLA-B27-negative patients had significantly higher levels of UA at baseline than HLA-B27-positive patients (*P* = 0.005). Pathologically, the percentage of globally sclerotic glomeruli in HLA-B27-negative patients was significantly higher than that in HLA-B27-positive patients (*P* = 0.02). All other baseline characteristics, including biomedical tests, renal pathological features, and initial therapies for AS, were comparable between the two groups (Table 1).

**Table 1:** Baseline demographic and clinical characteristics of HLA-B27-positive and HLA-B27-negative patients with secondary IgAN.

	**HLA-B27-positive (n=64)**	**HLA-B27-negative (n=7)**	**P value**
Male gender	47 (73.44%)	7 (100%)	0.19
Mean age (years), mean ± SD	36.53 ± 1.198	38.29 ± 4.352	0.66
Family history of ankylosing spondylitis	19 (29.6%)	0 (0.00%)	0.18
Disease duration since symptoms of AS (years), mean ± SD	12.38 ± 2.037	6.286 ± 1.686	0.33
Disease duration since diagnosis of IgAN (months), mean ± SD	61.87 ± 6.088	55 ± 17.61	0.71
Hypertension	30 (46.88%)	3 (46.68%)	1.00
Diabetes	9(14.1%)	0(0%)	0.588
Obesity	8(12.5%)	1(14.2%)	1.00
Axial manifestations			
Cervicalgia	8 (12.5%)	1 (14.2%)	0.43
Lumbalgia	44 (68.75%)	6 (85.71%)	0.67
Sacroiliac syndrome	35 (54.69%)	4 (45.31%)	1.00
Presence of peripheral arthritis	23 (35.94%)	0 (0.00%)	0.08
Extra-articular manifestations			
Psoriasis	4 (6.25%)	1 (14.29%)	0.41
Inflammatory bowel disease	1 (1.56%)	0 (0.00%)	1.00
Uveitis	2 (3.13%)	1 (14.29%)	0.27
Laboratory tests			
Proteinuria (g/d), mean ± SD	1.356 ± 0.1621	2.029 ± 0.5399	0.18
Serum creatinine (mg/dl), mean ± SD	1.143 ± 0.1649	1.197 ± 0.1442	0.92
GFR <60ml/min/l	2 (3.13%)	2 (3.13%)	0.25
Uric acid (umol/L), mean ± SD	354.2 ± 10.51	455.1 ± 42.18	0.005^*^
ESR (mm/h), mean ± SD	11.48 ± 1.698	16.63 ± 3.529	0.30
CRP (mg/L), mean ± SD	11.25 ± 2.588	10.83 ± 4.924	0.95
Renal histopathological lesions			
Glomeruli sclerosis (%)	14.66 ± 2.22	32.11 ± 9.36	0.02^*^
Segmental sclerosis (%)	5.91 ± 1.106	7.33 ± 2.73	0.67
Crescent (%)	3.40 ± 0.71	4.57 ± 3.46	0.62
IF/TA (Moderate-to-severe)	11 (17.19%)	3 (42.86%)	0.12
Untreated	15 (23.4%)	2 (28.57%)	0.67
Chinese traditional medicine	30 (46.8%)	2 (28.57%)	0.44
Sulfasalazine	30 (46.8%)	1 (14.29%)	0.12
Methotrexate	17 (26.6%)	1 (14.29%)	0.67
Leflunomide	12 (18.7%)	1 (14.29%)	1.00
Non-selective NSAIDs	14 (21.9%)	0 (0%)	0.33
COX-2 inhibitor	7 (10.9%)	0 (0%)	1.00
Steroids	30 (46.88%)	2 (28.57%)	0.45

* *P < 0.05. For qualitative variables as a percentage (%) and for quantitative variables as mean ± standard deviation. P values of univariate comparisons of baseline characteristics between HLA-B27 positivity and HLA-B27 negativity are shown (χ2 tests or Fisher's exact tests used for categorical variables). GFR, glomerular filtration rate; IFTA, interstitial fibrosis and tubular atrophy, NSAIDs, Nonsteroidal anti-inflammatory drugs*.

### Treatment After IgAN Diagnosis

After IgAN diagnosis, the initial therapies for AS were all discontinued. NSAIDS and traditional Chinese medicines were avoided in all patients because of their potential nephrotoxicity. During the follow-up period, 10 patients (15.6%) developed renal function decline despite the standard therapy for IgAN. Of these 10 patients, five were treated with Tripterygium Wilfordii Hook, four with oral prednisone, and four with leflunomide. In the remaining 61 patients, 30 were treated with Tripterygium Wilfordii Hook, 25 with leflunomide, and 15 with oral prednisone. All patients received angiotensin receptor blockade.

### Association Between HLA-B27 and Renal Function Decline

**Figure 1:**
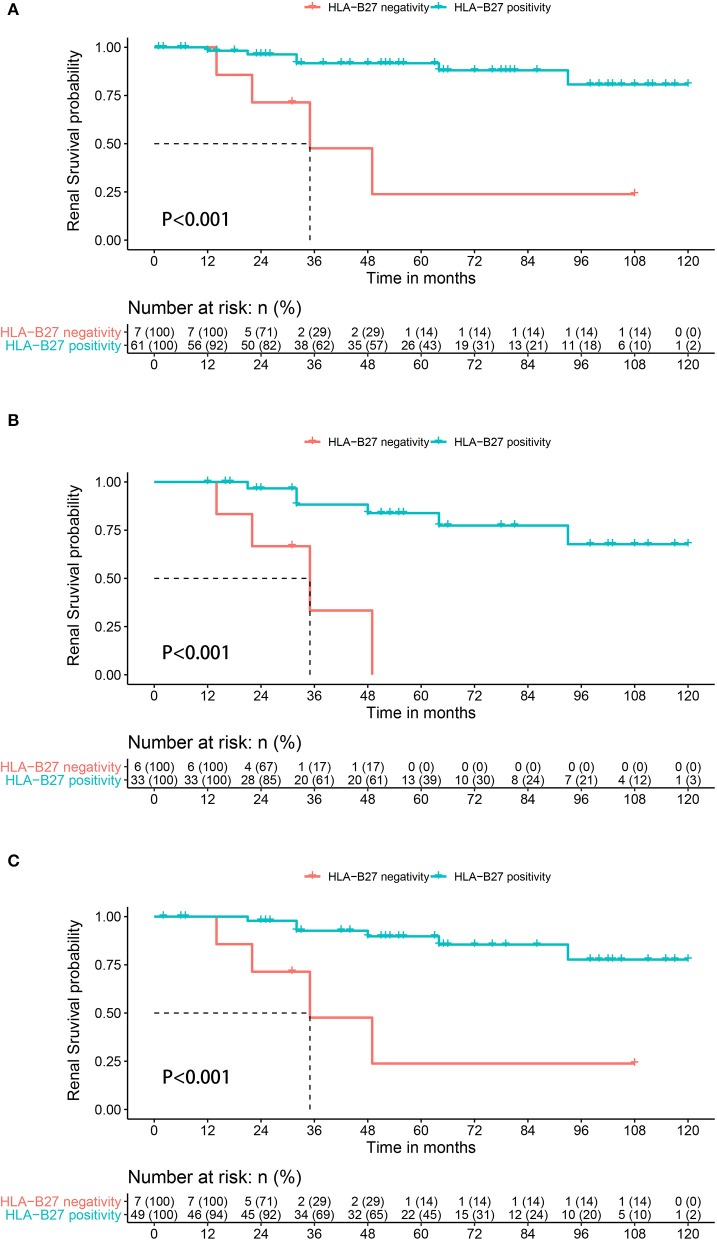
Kaplan-Meier survival plots for renal function decline during follow-up depending on the presence or absence of HLA-B27 in AS patients with IgAN **(A)** whole population, **(B)** in the subgroups of patients with proteinuria at >1.0 g per day, and **(C)** in the subgroups of patients with interstitial fibrosis and tubular atrophy.

Altogether, 10 patients met the definition of renal function decline during a median follow-up period of 2.8 years (IQR 2.0–4.1 years). Of these 10 patients, renal functions in five patients declined with a mean eGFR decline of 9.67 mL/min/1.73 m^2^ per year during the median follow-up period of 1.9 years, and dialysis was initiated in three patients; two patients had progressed to kidney transplantation at the last follow-up. There were four (57.1%) and six (9.0%) events occurring in the HLA-B27-negative and HLA-B27 positive groups, respectively. The probability of renal function decline in HLA-B27-negative patients was significantly higher than in HLA-B27-positive patients (*P* < 0.001) (Figure 1A).

Clinical characteristics, substantial proteinuria of > 1.0 g per day, and pathological marker IF/TA independently predicted a poor renal outcome in idiopathic IgAN (9). Six HLA-B27-negative and 31 HLA-B27-positive patients were included in the subgroup with substantial proteinuria, of whom renal function decline occurred in 10 patients. Three patients in the HLA-B27-negative group and four patients in the HLA-B27-positive group had renal function decline. The probability of renal function decline in HLA-B27 negative patients was also significantly higher than that in HLA-B27 positive patients (*P* < 0.001) (Figure 1B). In the subgroup of patients with ITFA, seven HLA-B27-negative and 49 HLA-B27-positive patients were analyzed. The HLA-B27-negative group had a significantly higher probability of renal function decline than HLA-B27-positive patients (*P* < 0.001) (Figure 1C).

### Associations Between HLA-B27 Negativity and Renal Function Decline

The general characteristics, clinical symptoms, laboratory results, and histological characteristics of the patients were included for statistical analysis. Univariable Cox regression analysis showed that factors significantly associated with renal function decline included HLA-B27 negativity (*P* < 0.001), Scr>1.24 mg/dl (*P* = 0.009), serum UA >421 umol/L(*P* = 0.048), focal segmental glomerular sclerosis (FSGS, *P* = 0.049), and moderate-to-severe IF/TA (*P* = 0.006), among which only HLA-B27 negativity was independently associated with renal function decline (HR 6.58; 95% CI 1.65 to 26.21; *P* = 0.008) (Table 2).

**Table 2:** Univariate and multivariate Cox regression analysis of prognostic factors for renal function decline in AS patients with secondary IgAN.

**Variable**	**No. (%) of participants (n=71)**	**Univariate analysis HR (95% CI)**	**P**	**Multivariate analysis HR (95% CI) ^**a**^**	**P**
Age					
≤ 31	31 (43.7)	1	-		
>31	40 (56.3)	0.63 (0.18-2.18)	0.466		
Gender					
Female	18 (25.3)	1	-		
Male	53 (74.6)	0.69 (0.12-1.63)	0.432		
Family history of AS					
No	49 (69.0)	1	-		
Yes	22 (31.0)	0.61 (0.13-2.89)	0.532		
Hypertension					
No	38 (53.5)	1	-	1	-
Yes	33 (46.4)	4.38 (0.93-20.67)	0.062	3.86 (0.78-19.16)	0.098
Diabetes					
No	62 (87.3)	1	-		
Yes	9 (12.7)	0.73 (0.09-5.80)	0.762		
Obesity					
No	62 (87.3)	1	-		
Yes	9 (12.7)	2.31 (0.48-11.2)	0.297		
HLA-B27					
Negativity	7(9.9)	1	-	1	-
Positivity	64 (90.1)	8.68 (2.41-31.29)	0.001	6.58 (1.65-26.21)	0.008
Proteinuria					
≤ 1g/d	28 (39.4)	1	-		
>1g/d	43 (60.6)	50.42 (0.29-8882)	0.137		
Serum creatine					
≤ 1.24mg/dl	57 (80.3)	1	-		
>1.24mg/dl	14 (19.7)	5.30 (1.53-18.35)	0.009		
Glomeruli sclerosis					
No	18 (25.3)	1	-		
Yes	53 (74.6)	1.84 (0.38-8.84)	0.447		
Serum UA					
≤ 421umol/L	58 (81.7)	1	-		
>421umol/L	13 (18.3)	3.50 (1.01-12.14)	0.048		
FSGS					
Negative	33 (46.4)	1	-		
Positive	38 (53.6)	4.77 (1.01-22.55)	0.049	4.08 (0.78-21.26)	0.095
IF/TA (Moderate-to-severe)					
No	57 (80.3)	1	-		
Yes	14 (19.7)	5.88 (1.66-20.87)	0.006		
Steroids					
No	45 (63.4)	1			
Yes	26 (36.6)	1.09 (0.31-3.87)	0.896		
Leflunomide					
No	46 (64.8)	1			
Yes	25 (35.2)	2.53 (0.69-9.27)	0.179		
TWHF					
No	29 (40.8)	1			
Yes	42 (59.2)	0.48 (0.14-1.68)	0.249		

AS, ankylosing spondylitis; HR, hazard ratio; CI, confidence interval; UA, uric acid; FSGS, focal segmental glomerular sclerosis; IT/FA, interstitial fibrosis and tubular atrophy; TWHF, Tripterygium Wilfordii Hook F.

a *The final model selection was carried out by a backward step-down selection procedure with the Akaike information criterion. Only covariates with a P < 0.10 in univariate analysis were included*.

## Discussion

A history of mucosal infections is characteristic of typical AS and IgAN patients, in whom the clinical symptoms emerge about several weeks postinfection (10). Meanwhile, IgA immune complex was detected in some AS patients, which is consistent with the proposed mechanism of IgAN in which circulating immune complexes lead to mesangial IgA deposition and renal tissue damage (11, 12). Therefore, nephropathy appears to be involved in the disease spectrum of AS. Wu et al. estimated renal involvement in AS patients to be over 20%, suggesting that secondary IgAN in AS patients may often be overlooked (13). To the best of our knowledge, ours is the first retrospective study and involves the largest cohort of AS patients with secondary IgAN to describe the clinical characteristics of AS patients with secondary IgAN based on the status of HLA-B27. In addition, we demonstrated that HLA-B27 negativity was associated with a higher probability of renal function decline in AS patients with secondary IgAN during follow-up periods.

In our study, the occurrence of HLA-B27 negativity was 9.5%, which is significantly lower than the 20% reported in Chinese population (14). HLA-B27-negative patients in our series were all male, which is consistent with male predominance in the study of Wu et al. (13), who reported that male gender was a risk factor for renal involvement in Chinese AS patients. However, univariate analysis of our study failed to indicate that gender was related to renal function decline.

Lee et al. and Azevedo et al. reported no difference in frequency of proteinuria and hematuria between HLA-B27-positive and -negative patients (15, 16). In our study, the severity of hematuria, proteinuria secretion, and renal function were also similar between the two groups. Arévalo1 et al. showed that HLA-B27 negativity was associated with a higher frequency of extra-articular manifestations, while HLA-B27 positivity was associated with earlier disease onset and a higher family-history tendency (17). We did not find significant differences in extra-articular manifestations, the duration of AS, or family history between the two groups in our series, probably due to the relatively small sample size. Increased serum UA has been shown to be a risk factor of renal involvement in Chinese AS patients (13). Indeed, the level of UA in HLA-B27-negative patients was higher than that in HLA-B27-positive patients in our study. Increased UA and declined renal function promote each other, forming a vicious cycle. The higher level of UA at presentation in HLA-B27-negative AS patients may suggest a downward trend of renal function during the follow-up period.

Marion at al. demonstrated that renal impairment (GFR <60) was associated with HLA-B27 positivity in their spondyloarthritis patients (18). However, they did not report whether HLA-B27 gene was related to the progression of renal damage in AS patients. In our study, the proportion of impaired renal function at baseline remained similar in both groups. Interestingly, HLA-B27-negative patients had a higher probability of developing renal function decline during the follow-up period. In the subgroup of patients with substantial proteinuria or IF/TA, which is believed to be linked to poor renal outcome in idiopathic IgAN, HLA-27 negativity was further confirmed as an indicator of renal function decline. Finally, potentially significant and clinically relevant predictors were analyzed in our multivariable Cox regression analysis, and the results showed that HLA-B27 negativity was an independent factor related to renal function decline. HLA-B27 negativity and positivity represent two distinct clinicopathological conditions in AS patients. Besides HLA-B27, other genetic factors revealed by genome-wide association studies (GWAS) were also responsible for the final clinical phenotype of AS. Surprisingly, risk alleles CARD9 (caspase recruitment domain-containing protein 9), which is known to promote the activation of NF-κB in macrophages, and TNFRSF13 (tumor necrosis factor ligand superfamily member 13), which induces IgA class switching, were both implicated in both IgAN GWAS and AS GWAS. The two risk alleles directly associate with the maintenance of the intestinal epithelial barrier and response to mucosal pathogens (10, 19, 20). We hypothesized that CAPD or TNFRSF13 conferred HLA-B27-negative AS and secondary IgAN patients with higher susceptibility to ESRD through the mediation of inflammatory pathways.

Our study has some limitations. First, recall bias may result from questioning patients about symptoms related to AS. In addition, we did not record and compare the activity index of AS patients between the two groups. Finally, the numbers of patients with renal function decline and HLA-B27-negative patients were relatively small, and therefore the implications and outcomes should be interpreted with caution.

In summary, this study demonstrated that the absence of HLA-B27 is related to a higher level of uric acid and a higher percentage of globally sclerotic glomeruli. In addition, HLA-B27 negativity was significantly associated with renal function decline during follow-up. These findings may remind clinicians to pay more attention to those HLA-B27-negative AS patients with secondary IgAN. Renal biopsy is advisable in AS patients with renal abnormalities to facilitate diagnosis and treatment. Urinalysis and renal function testing should be performed routinely, whether or not AS shows a controlled disease activity.

## Data Availability Statement

The raw data supporting the conclusions of this article will be made available by the authors, without undue reservation, to any qualified researcher.

## Ethics Statement

The studies involving human participants were reviewed and approved by Institutional Review Board of Jinling Hospital. Written informed consent for participation was not required for this study in accordance with the national legislation and the institutional requirements.

## Author Contributions

All authors listed have made a substantial, direct and intellectual contribution to the work, and approved it for publication.

### Conflict of Interest

The authors declare that the research was conducted in the absence of any commercial or financial relationships that could be construed as a potential conflict of interest.
